# Factors affecting resilience in Namibian children exposed to parental divorce: a Q-Methodology study

**DOI:** 10.3389/fpsyg.2023.1221697

**Published:** 2023-08-28

**Authors:** Janine Van Schalkwyk, Shelene Gentz

**Affiliations:** Department of Psychology and Social Work, University of Namibia, Windhoek, Namibia

**Keywords:** children, divorce, family instability, Namibia, protective factors; Q-Methodology, resilience

## Abstract

**Introduction:**

Divorce is a contributor to family instability within sub-Saharan Africa, and specifically within Namibia, an increasing number of children are exposed to its impact. However, not all children react uniformly to the impact of parental divorce, and many children may be resilient. Understanding what promotes resilience in children post-divorce in African contexts is vital, given the unique socio-cultural context. Therefore, this study aimed to understand how some children are capable of resilience despite exposure to parental divorce in Namibia.

**Methods:**

A multiple case study design was employed to assess the lived experiences of children aged 9–12 post-parental divorce in Windhoek. Using the Child and Youth Resilience Measurement (CYRM-12) scale, 24 children exposed to parental divorce were screened for resiliency. The Q-Methodology, with visual material, was utilized with a sub-sample of 12 children who scored high on the CYRM (50% girls, mean age = 11) to eliminate some of the challenges associated with gathering qualitative data from younger children. The PQ Method 2.35 software program was used for data analysis.

**Results:**

By-person factor analysis identified four statistically significant profiles. A third (33%) of participants loaded on a factor emphasizing “quality parent-child relationships” and a further 33% emphasizing “effective parent conflict resolution.” The final two factors emphasized “healthy school attachment” (17%) and “strong community attachment” (17%). All children emphasized a stable, loving familial environment, and frequent visitation with the non-custodial father.

**Discussion:**

Our findings suggest that multiple social ecologies nurture resilience in children exposed to parental divorce in Namibia. Support should be extended beyond the perimeters of the nuclear family, and relationships with extended family members, peer groups, school, and the wider community can play an important role in children's adjustment. The study highlights the importance of contextually grounded resilience as some factors that are emphasized for children from more Western communities do not reflect as strongly in the results of this study. Other factors, including a stronger reliance on community and factors such as the school, peers, and extended family members, may play a bigger role in child resilience post-divorce in Namibia.

## 1. Introduction

The family unit remains central to the optimal development of children, and, as such, the presence of divorce, a contributor to family instability (Clark and Brauner-Otto, 2015), constitutes a risk to children and youth. However, despite research findings on the negative consequences, not all children react uniformly to divorce (Brand et al., 2019), and many have been able to thrive (Kelly and Emery, 2003; Ruschena et al., 2005). Divorce is a global phenomenon, significantly affecting all lives connected to the breakdown of a marriage. Though accurate divorce rates have proven difficult to calculate (Amato, 2010; Kennedy and Ruggles, 2014), it is estimated that 50% of all marriages will lead to divorce (Anderson, 2014). In Namibia, the majority of marital unions and divorce occurrences are still conducted within African customary laws (Republic of Namibia Annotated Statutes, 2009). Hence, civil registration of marriages and divorces is limited, making statistical deductions and comparisons regarding divorce challenging. However, in 2015, The Namibian newspaper reported that divorce remained one of the primary societal problems in Namibia (The Namibian, 2016).

Several studies have reported poor adjustment of children post-divorce. In a meta-analysis of 54 studies spanning almost 30 years, Auersperg et al. (2019) found evidence of a consistent risk of a variety of mental health conditions post-divorce. This is confirmed by longitudinal research that found higher rates of anxiety/depression, and antisocial behavior in children whose parents divorced compared to those who remained married, both at the time of the divorce and several years later (Strohschein, 2005). Despite these findings, Karela and Petrogiannis (2020) argue that the consequences of divorce remain inconclusive and may depend on the presence of particular risk and protective factors. Some of the more consistent risk factors associated with a negative outcome have been linked to the parental subsystem. In a meta-analytic study, e.g., van Dijk et al. (2020) found that interparental conflict and negative parental behaviors had a negative effect on child adjustment. Indeed, with younger children, Karela and Petrogiannis (2020) found that children who exhibited higher levels of resilience experienced less parental stress and that there was a more supportive relationship between the parents. Similarly, for adolescents, increased internalizing problems are reported with negatively engaged parents (Rejaän et al., 2022), whereas adolescents whose parents exhibit a cooperative pattern show the least amount of internalizing and externalizing problems. Indeed, parental support has been linked to overall better outcomes for children supporting the notion by Fergus and Zimmerman (2005) that secure attachment to either one or both parents tends to mitigate the subsequent effects of exposure to family adversity and hence, promotes resilience in the child. The role of parental involvement, including the role of the father, has been found to have a positive impact on children's adjustment (Amato, 1991). However, despite the post-modernistic shift in research focusing on the development and identification of individual and familial strengths, the studies that investigate positive outcomes as well as normal development in individuals living in alternative familial structures such as those created by the dissolution of a marriage are still limited, particularly in non-Western contexts. Research continues to portray a conservative perspective by focusing on the detrimental influences of parental divorce on children's capacity for healthy adjustment (Bernardi and Radl, 2014; Arkes, 2015) and remains a concern (Mohi, 2014). The need to explore processes that enable positive adjustment to multiple transitions amidst marital dissolution remains.

The past decade has brought about a budding interest in the positive aspects of human functioning and adaptability, especially in the context of adversity such as marital dissolution (Seligman and Csikszentmihalyi, 2000). Furthermore, scholars have been exploring the notion of beneficial parental divorce as children from high-conflict households are more inclined to experience behavioral and psychological problems (Amato and Kane, 2011). Moreover, conservative notions of divorce have led many parents to remain in unhealthy, high-conflict marriages out of fear of the detrimental impact that exposing children to a single-parent family might have and disregarding that this could pose more harm than good (Gadoua, 2008). For example, Lindsey et al. (2009) note that in terms of family interrelations, the development of the child's capacity for social interactions and their sense of security is negatively impacted. Hence, some have argued that a divorce may become an avenue for peace of mind, growth, and a chance for children to thrive (Chavez, 2010). Furthermore, divorce may provide a child with an environment characterized by fewer stressors, which, in turn, will facilitate normal development (Amato et al., 2011).

Children are forced to grow up and function in an increasingly stressful world. Therefore, it becomes unrealistic to argue that they can be protected from experiencing negative events. Over the last 50 years, resilience research involving children and families has aimed to investigate the health-enhancing capacities, the presence of resources within an individual, familial, or societal context as well as the specific developmental pathways of those most vulnerable (Pedro-Carroll, 2001; Kelly and Emery, 2003; Theron and Theron, 2010; Ungar, 2012). The concept of resilience refers to the process characterized by exposure to significant risk and a subsequent positive developmental outcome amidst that exposure (Rutter, 2006). Building on these definitions, resilience is now increasingly conceptualized as an interactive process—between the individual and their environment as well as among protective and risk factors—and not as a fixed individual attribute. Resilience is, therefore, increasingly not viewed as a static trait. The ecological systems theory explores the interrelationship between the individual and their unique environments to determine possible developmental impacts thereof on the child (Bronfenbrenner, 1986; Garmezy, 1991; Garbarino, 1995). Garmezy's (1991) triadic model of resilience explained the dynamic interaction between protective and risk factors on three levels, namely the individual, the family, and the environment. Furthermore, the model continues to highlight resilience as a means of empowering individuals to shape and be shaped by their environment. Similarly, the interactive ecological-transactional model of development emphasizes how development and adaptation are influenced by the interaction among different contexts, such as culture, neighborhoods, and family (Cicchetti and Lynch, 1993). Thus, the degree of resilient features in an individual depends on the extent to which environmental factors are able to nurture the capacity for resilience. Resilience can be seen as context dependent; elements in the child's surroundings need to support and nurture resilience for the child to experience improvements in their wellbeing.

Mainstream resilience research continues to stem from a Eurocentric epistemology, placing emphasis on factors of resilience characteristics of the mainstream population and their accompanying definition of healthy adjustment (Ungar, 2004, 2008; Boyden and Mann, 2005). As a result, limited investigation has been conducted into the relevance of resilience to non-Western world cultures, in which the necessary resources required for survival may vary compared to those available to Western populations (Ungar, 2008). Similarly, Masten (2014) emphasized the need to understand what happiness and wellbeing mean in different contexts and experiences. For example, up to 37% of children in Namibia do not live with a biological parent [The Nambia Ministry of Health Social Services (MoHSS) ICF International, 2014]; this highlights the role that extended and informal care systems may play in children's lives. According to Cowen (1994), depending on different situations, there may be different pathways to resilience. While some features of healthy adjustment might be relevant to various populations, the significance of each varies when cultural and contextual differences are considered, emphasizing the idiosyncratic nature of various survival processes (Ungar, 2008).

Therefore, the present study aimed to explore the lived experiences of resilience in children, post-parental divorce, within middle childhood, in one African context, Namibia. The study seeks to identify and understand the protective factors that nurture the capacity to adjust and thrive, post-parental divorce. As such, as we are attempting to understand protective factors at different levels and how the child interacts with them, we consider resilience to be a process. Middle childhood, which typically includes the years from 9 to 12, is an important developmental phase for children cognitively, socially, and emotionally, and for the development of their self-concept (Louw, 1998). Children in this developmental phase may be especially vulnerable to the effects of parental divorce as the development of constructive social relationships and self-esteem occurs during this stage. The family continues to play a crucial role in the socialization of the child during middle childhood. Furthermore, research on parental divorce continues to be predominantly conducted from the perspective of adolescents and adults, emphasizing the need for studies to explore younger children's perspectives on their experiences of parental divorce (Maes et al., 2012). This is especially important as post-divorce adjustment tends to be mediated primarily by the child's perceptions and experiences of the divorce event (Maes et al., 2012).

## 2. Methods and materials

### 2.1. Q-Methodology

The current study uses a Q-Methodology, considered by most researchers to be a mixed-method, as data are collected qualitatively using a small number of participants but analyzed using quantitative methods (Bashatah, 2016). Q-Methodology is, therefore, a hybrid method that contains elements of interviewing, thematic analysis, and factor analysis (Størksen et al., 2012). Q-Methodology was primarily designed for the purpose of investigating and categorizing patterns of individual perspectives and lived experiences related to a specific topic by conducting rigorous quantitative analysis (McKeown and Thomas, 2013). Moreover, by analyzing these individual responses, the researcher is able to extract rich data. In this manner, it becomes possible to explore subjectivity quantitatively. Q-Methodology seeks to explore and interpret various viewpoints that exist within the target population (Ward, 2010). Using a multiple case study design, the study seeks to provide a more in-depth comprehension of the multiple facets involved in child resilience after exposure to divorce and a better understanding of the differences and similarities between participants.

### 2.2. Sample and sampling procedure

The participants of this study were selected using purposeful sampling (Yin, 2011), with participants from schools in Windhoek selected based on their availability and willingness to participate in the study. However, of the total of eight schools approached, only two agreed to participate, largely due to the COVID-19 pandemic. Children between the ages of 9 and 12 whose parents had been divorced for 2–4 years were invited to participate in the study. Due to the sensitive nature of the topic, all recruitment was done by the schools, which were provided with all the necessary information. Children undergoing therapy and exposed to multiple parental divorces were excluded from the study.

Overall, 24 children (between the ages of 9 and 12) were part of the data collection process. However, as this study was specifically interested in resilience, only the data from 12 children scoring high on the resilience measure were included in the final study (Table 1). In a Q-Methodology study, participants are viewed as variables; hence, it is not necessary for the number of participants to be excessively large (Webler et al., 2009). Of the 12 participants, 50% were girls, and the mean age was 11 years. The majority of the participants (41.7%) were in grade 5, while the remaining participants were in grades 3, 4, and 6. The home languages reported by the participants were mainly English (33.3 %), with a few of the participants being Afrikaans, Khoekhoegowab, and Oshiwambo speakers. The average number of years since the parental divorce was 3 years.

**Table 1:** Socio-demographic data (*n* = 12).

	***n***	**%**
**Age (years)**		
9	2	16.7
10	3	25.0
11	5	41.7
12	2	16.7
**Sex**		
Male	6	50.0
Female	6	50.0
**Grade**		
Grade 3	2	16.7
Grade 4	3	25.0
Grade 5	5	41.7
Grade 6	2	16.7
**Custodial parent**		
Mother	12	100.0
**Home language**		
Oshiwambo	2	16.7
Afrikaans	3	25.0
Nama/Damara	3	25.0
English	4	33.3
**Time since parental divorce**		
2 years	7	58.3
3 years	4	33.3
4 years	1	8.3

#### 2.2.1. The Child and Youth Resilience Measurement

The Child and Youth Resilience Measurement (CYRM) questionnaire was administered to all 24 participants to screen for resilience (Ungar and Liebenberg, 2011), with higher total scores indicating the presence of more resilience components. By incorporating upper and lower half scoring, 12 participants were identified as having more resilience components than their counterparts. This upper half group had a mean score of 47 (range: 43–51), whereas the lower half group had a mean score of 26 (range: 26–35).

### 2.3. Instruments

#### 2.3.1. Socio-demographic questionnaire

Each participant completed a short socio-demographic questionnaire (age, sex, and home language of each participant).

#### 2.3.2. The Child and Youth Resilience Measurement (CYRM-12)

The CYRM-12, a 17-item scale on a 3-point Likert scale, was administered to all 24 participants to screen for resiliency (Ungar and Liebenberg, 2011). The CYRM-12 is a measure of individual, relational, and communal resources available to individuals that may enhance resilience. The CYRM-12 demonstrated a good fit to the Rasch model (α = 0.82) and is applicable across diverse cultures and contexts, including children from South Africa (van Rensburg et al., 2019).

#### 2.3.3. The Q-set

To resolve some challenges of gathering qualitative data from younger children, the Q-Methodology (Brown, 1980) with visual material was utilized. Participants who take part in a Q-Study are exposed to a set of cards containing either subjective statements or visual images related to the research topic. In this method, participants rank several statements about the topic in relation to other statements. The statements are referred to as a Q-set.

**Figure 1:**
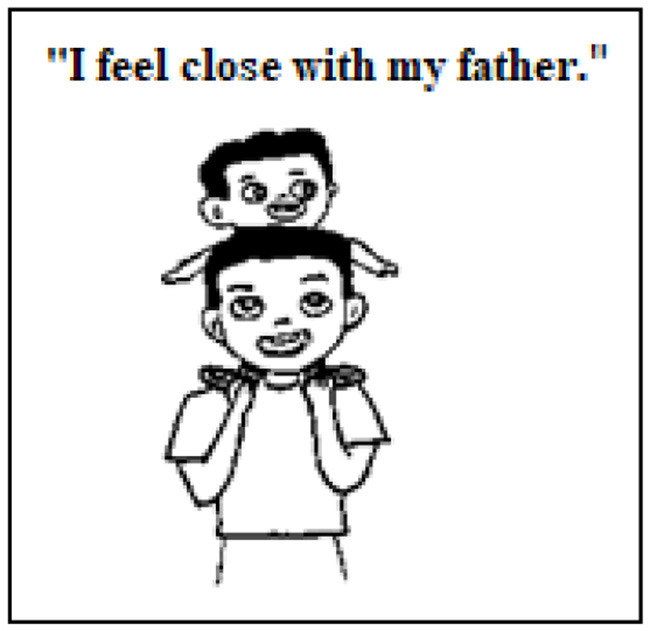
Example of a card with visual material used during the study.

The first step in the Q-Methodology involved the development of an initial concourse. For this particular study, the initial concourse was developed through an extensive literature review of possible indicators of resilience among children exposed to parental divorce (Brown, 1980; Van Exel and de Graaf, 2005). Various databases were searched using terms related to resilience, divorce, and its impact, as well as risk and protective factors related to the individual, family, and community. It was important to ensure that both intra-familial and extra-familial factors could be examined. Furthermore, reference lists of key literature were scanned to further expand the search. For the second step, a Q-sample (a set of statements) related to parental divorce and instances of resilience was generated from this initial concourse and distilled. It was important that all statements identified be representative of the different aspects of the broader concourse and that there were statements with which participants could agree as well as disagree (Coogan and Herrington, 2011). Piloting (see below) these statements was an important step to ensure that an extensive range of coverage was achieved. Once statements were developed, they were subdivided into various categories (e.g., familial protective factors and extra-familial protective factors). These categories function to ensure that all sub-aspects of the topic have been included and that these statements do not display favoritism toward some aspects over others (Coogan and Herrington, 2011). The subjective viewpoints of each participant could only be discovered if all possible areas were explored. Furthermore, the total number of statements had to make it possible to produce different viewpoints from the self-reference of the participant (Thorsen, 2006). Once the Q-sample is generated, it is generally known as a Q-set and is placed on cards for participants to effectively sort through the statements. Visual pictures, designed by the first author, were used together with the Q-set to provide children with guidance on how to verbally express their emotions (Figure 1). The complexity and number of statements depend on the cognitive and developmental level of the participants, but the sample of statements normally ranges between 40 and 80 (Alderson et al., 2018). Considering both the age and developmental level of participants, this study contained 40 statements to ensure that children had the mental capacity to maintain focus throughout the sorting process.

### 2.4. Pilot study

A pilot study in the form of a focus group discussion was conducted with six participants of both sexes, aged 9, in order to test the clarity and general comparability of the visual statements for the participants. Participants were also asked whether they felt any other aspects of their post-divorce experience should be included. A group size of six participants was deemed optimal (de Leeuw, 2011) due to the age of the participants (Morgan, 1997), and group discussions did not exceed 30 min. All participants communicated a clear understanding of both the instructions and the content of each statement; hence, no major alterations were made to the set of statements.

### 2.5. Procedure

Once participants were identified, contact with the legal guardian was initiated. All required documentation, including an information leaflet, parental consent, and participant assent forms, was provided and completed before the data collection process began. Each school provided a vacant classroom and indicated a suitable time for data collection. Data were collected by the first author (JV) in the following order: CYRM-12 and the socio-demographic questionnaire, followed by the Q-sorting procedure. During the Q-sorting procedure, the children/participants were individually presented with the Q-set (set of statements) on individual cards together with a predesigned quasi–normal-shaped distribution grid developed for the sorting of the cards. Participants were invited to consider their parents' divorce and to sort each card in the distribution grid “in accordance with what is most like or unlike” their situation. The distribution grid placed the most agreeable statements (most like) on one side and the most disagreeable statements (most unlike) on another side. If a statement was found more on the right side of the grid, the participant's sorting “agreed” more with the statement and vice versa for statements found more on the left side. Rankings ranged from +3 (strongly agree) to −3 (strongly disagree). In order to make the sorting process easier for the participants, they were advised to create three piles of statements, namely those they agreed with, those they mostly disagreed with, and those they felt mostly neutral about (Coogan and Herrington, 2011). Thereafter, participants were instructed to place each pile of statements on the grid according to their level of agreement, working to fill the columns representing all the agreed upon statements until they were depleted. The same principle was applied for statements that were most disagreed with, but these were placed under the −3 column. The subsequent open spaces in the center of the distribution grid were then filled with all the statements the participant felt mostly neutral or uncertain about. Once all statements were placed on the Q-grid and the participant was satisfied with their sorting, it formed what is referred to as the Q-sort. The Q-sort reflected each participant's perspective and experiences surrounding the topic. This was recorded by a photograph for later quantitative analysis (McKeown and Thomas, 2013). The entire interview, together with the Q-sorting procedure, lasted for 40–60 min.

### 2.6. Data analysis

Descriptive statistics (socio-demographics) were analyzed using IBM SPSS Statistics for Windows, Version 21.0. All Q-sorts were plotted into the PQ Method 2.35 software program (Schmolck, 2014) and analyzed using by-person factor analysis (Watts and Stenner, 2005). Hence, participants correlate with others who display similar perspectives based on their Q-sorts (Valenta and Wigger, 1997). Intercorrelations between each individual Q-Sort were determined by computing a correlation matrix (Brown, 1980) using principal component analysis (Militello and Benham, 2010).

This resulted in the identification of factors representing clusters of participants who share the same perspectives or experiences in relation to the topic—in this case, displays of resilience post-parental divorce (Van Exel and de Graaf, 2005; Akhtar-Danesh et al., 2008). By using the correlation matrix, different sorts were grouped into eight unrotated “factor” groups of participants who share similar viewpoints, which is the maximum number of factors that can be extracted using the PQMethod program. In contrast to the R-method of factor analysis which groups variables, Q-Method analyses the data by grouping participants (McKeown and Thomas, 2013).

Factors that have eigenvalues >1.00 were deemed significant, explaining a significant amount of variability within the data (Watts and Stenner, 2005). Eigenvalues <1.00 are considered too weak and do not explain a significant amount of variance within the data. In addition, the factor must have a minimum of two significant participant loadings. Using the Varimax technique, four factors (Factors 1–4) had eigenvalues >1.00 and hence warranted further exploration (Table 2).

**Table 2:** Four-factor solution following Varimax rotation.

**Q-Sort ID**	**Factor 1**	**Factor 2**	**Factor 3**	**Factor 4**
P1	0.8348^*^	−0.0231	0.1266	0.2993
P2	0.2595	0.1192	0.2772	0.8648^*^
P3	0.2975	−0.0438	0.0069	0.9078^*^
P4	0.8917^*^	0.0661	0.1805	0.2633
P5	0.1355	0.2379	0.4893^*^	0.6941^*^
P6	0.8816^*^	0.0164	0.0956	0.1422
P7	0.1287	−0.1204	0.9197^*^	0.2100
P8	−0.1147	0.9083^*^	−0.0657	0.1394
P9	0.1488	0.9062^*^	−0.0809	0.0211
P10	0.5423^*^	0.0994	0.1978	0.6732^*^
P11	0.8977^*^	−0.0082	0.2011	0.2084
P12	0.2982	−0.0985	0.8949^*^	0.1697
% explained variance	31	15	18	24
Number of participants loading	4	2	2	4

^*^Significance at a p-value of <0.01.

The rotated factors comprise 88% of the total variance of the study, where Factor 1 represents 31%, Factor 2 represents 15%, Factor 3 represents 18%, and Factor 4 represents 24%. For Factor 1, four participants were loaded onto the factor with a significance level of a *p*-value of <0.01. On Factor 2, two participants were loaded at a significance level of a *p*-value of <0.01. Factor 3 also comprised two participants with a loading at a *p*-value of <0.01 significance level and Factor 4 had four participants with a loading at a significance level of a *p*-value of <0.01. No participant was loaded on more than one factor. The correlation between the final four factors that were extracted is shown in Table 3. Factors 1 and 4 presented the highest correlation (0.56), without any participants loading on both factors.

**Table 3:** Correlation between factor scores.

	**Factor 1**	**Factor 2**	**Factor 3**	**Factor 4**
Factor 1	1.0000	0.0381	0.3973	0.5591
Factor 2	0.0381	1.0000	−0.1601	0.1366
Factor 3	0.3973	−0.1601	1.0000	0.4259
Factor 4	0.5591	0.1366	0.4259	1.0000

### 2.7. Research ethics

Permission for the study was obtained from the University of Namibia's Research and Ethics Committee (UREC) and the Khomas Regional Council (Directorate of Education, Arts, and Culture). In each case, the primary caregiver provided written informed consent, and written assent was sought from each participant. Participants were continuously reminded of their right to withdraw from the study with no resulting negative consequences. Specific signs indicative of the child's wishes to withdraw were agreed upon. Personal information remained confidential unless there was a risk of harm. To maintain anonymity, each participant was assigned a unique code for the questionnaires and answer sheets. To minimize harm, the researcher was mindful of possible effects on the child throughout the process. Participants and their parents had access to counseling services when it was required. Data obtained from participants will be stored for 5 years in a lockable cabinet to which only the researchers have access.

## 3. Results

The following section describes each factor individually. The discussion of the factors will not follow a normal numerical pattern but will be based on where most participants are loaded. Therefore, Factor 1 (33% of participants) and Factor 4 (33%) will be discussed first, followed by Factor 2 (17%) and Factor 3 (17%).

### 3.1. Factor 1: quality parent–child relationships

The highest positive set of statements for participants that loaded on Factor 1 is shown in Table 4. Four participants (33%) were significantly loaded on Factor 1, two being Afrikaans speakers, one being an Oshiwambo speaker, and one being a Nama/Damara speaker. Three participants (75%) were male. Participants who loaded onto this factor identified statements emphasizing the positive quality of the relationship with their parents. Statements indicate that participants place emphasis on the quality of the relationship with both parents pre- and post-divorce and are also suggestive of a high value placed on transparency and effective communication within those relationships, as well as frequent contact with their father.

**Table 4:** Factor 1 most agree and disagree statements.

**Statement #**	**Most agree statements**	***z*-score**	**Grid position**
1	I feel close to my mother	1.748	3
2	I feel close to my father	1.748	3
3	My family loves me	1.601	3
16	I feel safe and loved at home	1.544	3
7	I see my father regularly	1.415	2
8	I felt close to my father before their divorce	1.166	2
9	I felt close to my mother before their divorce	1.166	2
17	I feel comfortable talking to my parents	1.166	2
20	My parents always talk to me and explain things I do not understand	1.166	2
19	My parents explained their decision to divorce to me	1.019	2
**Statement #**	**Most disagree statements**	***z*** **-score**	**Grid position**
33	We stayed in the same home after the divorce	−1.748	−3
32	I remained in the same school after the divorce	−1.748	−3
34	We stayed in the same neighborhood after the divorce	−1.748	−3

The two strongest agreed statements were “I feel close to my mother” and “I feel close to my father,” suggesting that these participants value a close, secure relationship with both their parents. Moreover, the next two highest ranked statements, “My family loves me” and “I feel safe and loved at home,” showed that these participants view their familial environment as overall safe and loving. Statement 6 (“I see my father regularly”), also among the top-ranked statements, indicates that these participants have regular contact with the non-custodial parent. Combined with a high rating of a close relationship with their father (statement 2), a positive father–child attachment is suggested. Also among the highest ranked statements are statement 17, 20 and 19 which highlight participants' positive rating of the quality of the parent–child communication.

For participants loading on Factor 1, the strongest disagreement was expressed with statement 33 (“We stayed in the same home after the divorce”), statement 32 (“I remained in the same school after the divorce”), and statement 34 (“We stayed in the same neighborhood after the divorce”). The statements collectively suggest that, while maintaining stability and familiarity is important for post-parental divorce adjustment, it was not perceived as an important factor for participants in this group.

### 3.2. Factor 4: effective parent conflict resolution and relationships

One-third of participants (33.3%) were significantly loaded on Factor 4 (*p* < 0.01), with two being English speakers, one being an Afrikaans speaker, and one being an Oshiwambo speaker. Furthermore, Factors 1 and 4 showed the highest correlation (0.56), without participant loadings on both factors. Participants who loaded significantly on this factor, rated statements highly that emphasized effective parental conflict resolution as well as their ability to maintain a civil and cooperative relationship post-divorce as important contributors to resilience after their parents' divorce (Table 5). Among the highest sorted statements were statement 4 “My parents get along well,” statement 13 “My parents fight less now than before the divorce,” and statements 11 and 12, indicating parents' ability to refrain from speaking negatively about each other in front of their children. These statements highlight the participants' shared belief that their parents' ability to resolve conflict civilly and cooperatively has contributed to the children's capacity to adjust post-divorce.

**Table 5:** Factor 4 most agree and disagree statements.

**Statement #**	**Most agree statement**	***z*-score**	**Grid position**
4	My parents get along well	1.770	3
11	My mother does not speak badly of my father in front of me	1.770	3
12	My father does not speak badly of my mother in front of me	1.77	3
13	My parents fight less now than before the divorce	1.524	3
10	My mother is happy to allow visits with my father	1.427	2
5	I feel close to my grandmother	1.180	2
3	My family loves me	1.044	2
**Statement #**	**Most disagree statement**	***z*** **-score**	**Grid position**
31	I feel close to my coach	−1.033	−2
24	I have fun at school	−1.136	−2
29	I feel close to my friend's parents	−1.18	−2
15	I see my grandfather often	−1.361	−3
25	I am happy to go to school	−1.499	−3
34	We stayed in the same neighborhood after the divorce	−1.77	−3
33	We stayed in the same home after the divorce	−1.77	−3

Furthermore, participants who loaded significantly on this factor, highly rated statement 10 “My mother is happy to allow visits with my father.” As all participants identified their mother as the custodial parent, this group of children was able to see their non-custodial parent (their father) on a regular basis, with cooperation from their mother. Noteworthy, is the fact that participants who loaded on Factor 4 sorted statements 33 “We stayed in the same home” and statement 34 “We stayed in the same neighborhood after the divorce” among the lowest positions (-3), much like those participants in the Factor 1 group (Table 5).

While Factors 1 and 4 share the highest correlation (0.56), some distinct differences between the two groups also occurred (Table 6). The statements listed in Table 6 are all three or more columns apart on the distribution grid. Participants who loaded on Factor 1 identified statement 20 “My parents always talk to me and explain things that I do not understand” and statement 19 “My parents explained their decision to divorce to me” as important compared to participants who loaded on Factor 4. This sorting ties into their shared perspective, which identified quality familial/parental–child relationships and transparent communication among the most important protective factors in nurturing their resilient capacities and ability to adjust after the divorce of their parents. As opposed to Factor 1, participants in Factor 4 rated statement 32 “I remained in the same school after the divorce” higher on the distribution grid (+1).

**Table 6:** Differing statements between Factors 1 and 4.

**Statement #**	**Statement**	**Factor 1 values**	**Factor 2 values**
20	My parents always talk to me and explain things that I do not understand	2	−1
32	I remained in the same school after the divorce	−3	1
26	I am happy and satisfied most of the time	−2	1
19	My parents explained their decision to divorce to me	2	−2

### 3.3. Factor 2: healthy school attachment

Two of the 12 participants (17%) significantly loaded on Factor 2 (*p* < 0.01), one Nama/Damara-speaking child, and one English-speaking child. Table 7 below presents the arrangement of statements by the participants who scored significantly on this factor. Participants identified healthy school attachment as an important element in their lives. Participants highly ranked statements 28 and 31, which signal a close relationship with their teacher and coach. Furthermore, a high ranking on statement 22 “Teachers at my school help and support me,” statement 39 “I feel safe and happy at school,” statement 24 “I have fun at school,” and statement 25 “I am happy to go to school” further suggests the importance placed on safety, happiness, and secure attachments at school.

**Table 7:** Factor 2 most agree and disagree statements.

**Statement #**	**Most agree statement**	***z*-score**	**Grid position**
28	I feel close to my teacher	1.766	3
31	I feel close to my coach	1.766	3
32	I remained in the same school after the divorce	1.766	3
22	Teachers at my school help and support me	1.475	3
39	I feel safe and happy at school	1.468	2
24	I have fun at school	1.178	2
25	I am happy to go to school	1.178	2
30	I participate in activities after school	1.178	2
**Statement #**	**Most disagree statement**	***z*** **-score**	**Grid position**
37	My friends and I follow the rules	−1.178	−2
29	I feel close to my friend's parents	−1.178	−2
36	I have enough food to eat every day	−1.475	−2
34	We stayed in the same neighborhood after the divorce	−1.766	−3
15	I see my grandfather often	−1.766	−3
35	I feel safe in my neighborhood	−1.766	−3

Among the lowest ranked statements are those that touch on socioeconomic conditions such as a satisfactory amount of food to eat every day (statement 36) and conditions surrounding the neighborhood within which the participants reside (statements 35 and 14). This result indicates that this group of children regarded these conditions as least contributing to their ability to adjust and thrive after their parents' divorce.

### 3.4. Factor 3: strong community attachment

Two participants (0.17%) loaded significantly on Factor 3, one Nama/Damara speaker and one Afrikaans-speaking child. Statements ranked highly by participants in this factor identified a close relationship with members of the community, extended family members, and parents of peers as an influential factor in dealing with the divorce of their parents (Table 8). Among the highest ranked statements were statements 5 and 6, which indicated a close relationship between the participants and their grandparents, as well as statement 29 (“I feel close with my friends' parents”). Statement 23 (“I have close friends”) was also among the highest placed statements and signals that participants in this group identified peer relationships as important.

**Table 8:** Factor 3 most agree and disagree statements.

**Statement #**	**Most agree statements**	**z-score**	**Grid position**
5	I feel close to my grandmother	1.678	3
23	I have close friends	1.678	3
29	I feel close to my friend's parents	1.678	3
6	I feel close to my grandfather	1.438	3
14	I see my grandmother often	1.359	2
1	I feel close to my mother	1.119	2
15	I see my grandfather often	1.119	2
16	I feel safe and loved at home	1.119	2
**Statement #**	**Most disagree statements**	**z-score**	**Grid position**
26	I am happy and satisfied most of the time	−1.119	−2
31	I feel close to my coach	−1.119	−2
33	We stayed in the same home after the divorce	−1.359	−2
34	We stayed in the same neighborhood after the divorce	−1.359	−2
25	I am happy to go to school	−1.359	−2
19	My parents explained their decision to divorce to me	−1.438	−3
20	My parents always talk to me and explain things that I do not understand	−1.438	−3
32	I remained in the same school after the divorce	−1.678	−3

Children who loaded on Factor 3 sorted statement 19 (“My parents explained their decision to divorce to me”) and statement 20 (“My parents always talk to me and explain things that I do not understand”) on the lowest range of the sort. The placement of these statements indicated the participants' shared perspective that transparent communication has not been an important factor when considering resources that nurture their resilience.

### 3.5. Distinguishing statements

Distinguishing statements refer to statements that are highly ranked on a specific factor in comparison to their rank on the other factors. They enable the researcher to understand the ways in which the extracted factors are unique. The four highest ranked distinguishing statements (statements 2, 17, 20, and statement 19) for Factor 1 indicate the value participants in this group place on a close attachment to one or both parents, together with a transparent and supportive communication (Table 9).

**Table 9:** Distinguishing statements for each factor.

**Statement No**.	**Distinguishing statement**	**Factor 1**		**Factor 2**		**Factor 3**		**Factor 4**	
		***Q*** **-sort value**	***z*** **-score**	***Q*** **-sort value**	***z*** **-score**	***Q*** **-sort value**	***z*** **-score**	***Q*** **-sort value**	***z*** **-score**
2	I feel close to my father	**3**	**1.75**	1	0.29	2	0.88	2	0.84
17	I feel comfortable talking to my parents	**2**	**1.17** ^*^	−1	−0.3	0	0.00	0	−0.14
20	My parents always talk to me and explain things that I do not understand	**2**	**1.17** ^*^	0	0.00	−3	−1.44	−1	−0.50
19	My parents explained their decision to divorce to me	**2**	**1.02**	0	0.00	−3	−1.44	−2	−0.87
18	I have rules set by my parents and they hold me to it	**1**	**0.83**	−2	0.59	−2	−0.96	0	0.00
28	I feel close to my teacher	−2	−0.81	**3**	**1.77** ^*^	0	−0.08	0	−0.21
31	I feel close to my coach	−1	−0.48	**3**	**1.77** ^*^	−2	−1.12	−2	−1.03
32	I remained in the same school after the divorce	−3	−1.75	**3**	**1.77** ^*^	−3	−1.68	1	0.18
22	Teachers at my school help and support me	0	−0.33	**3**	**1.48** ^*^	0	0.00	0	−0.18
39	I feel safe and happy at school	−1	−0.54	**2**	**1.47** ^*^	−1	−0.56	−1	−0.66
24	I have fun at school	−2	−0.89	**2**	**1.18** ^*^	−1	−0.24	−2	−1.14
25	I am happy to go to school	−3	−1.06	**2**	**1.18** ^*^	−3	−1.36	−3	−1.50
30	I participate in activities after school	0	−0.27	**2**	**1.18** ^*^	−1	−0.56	−1	−0.51
33	We stayed in the same home after the divorce	−3	−1.75	**1**	**0.30** ^*^	−3	−1.36	−3	−1.77
19	My parents explained their decision to divorce to me	2	1.02	**0**	**0.00**	−3	−1.44	−2	−0.87
5	I feel close to my grandmother	1	0.58	**−1**	**−0.30**	3	1.68	2	1.18
16	I feel safe and loved at home	3	1.54	**−2**	**0.88** ^*^	2	1.12	1	0.75
35	I feel safe in my neighborhood	−2	−0.75	**−3**	**−1.77**	−1	−0.56	−2	−0.91
23	I have close friends	0	−0.36	0	−0.29	**3**	**1.68** ^*^	1	
29	I feel close to my friend's parents	−2	−0.86	−2	−1.18	**3**	**1.68** ^*^	−2	
6	I feel close to my grandfather	1	0.48	−1	−0.3	**3**	**1.44**	−1	
14	I see my grandmother often	−1	−0.72	−1	−0.57	**2**	**1.36** ^*^	−1	
15	I see my grandfather often	−1	−0.72	−3	−1.77	**2**	**1.12** ^*^	−3	
20	My parents always talk to me and explain things that I do not understand	2	1.17	0	0.00	**−3**	**−1.44**	−1	
4	My parents get along well	1	0.58	1	0.01	1	0.32	**3**	**1.77** ^*^
11	My mother does not speak badly about my father in front of me	1	0.29	1	0.59	1	0.56	**3**	**1.77** ^*^
12	My father does not speak badly about my mother in front of me	1	0.29	1	0.59	1	0.56	**3**	**1.77** ^*^
27	I enjoy playing and having fun	0	−0.21	−2	−0.59	−1	−0.80	**2**	**0.79** ^*^
26	I am happy and satisfied most of the time	−2	−0.80	−2	−0.88	−2	−1.12	**1**	**0.66** ^*^
32	I remained in the same school after the divorce	−3	−1.75	3	1.77	−3	−1.68	**1**	**0.18** ^*^

All values in bold are significant at p <0.05; ^*^ values are also significant at p <0.01.

The highest ranked statements (statements 28, 31, 32, 22, and 39) for Factor 2 indicated that children who loaded on this factor reported a close attachment with their school and its personnel as a protective factor. This group of participants did not agree that feeling safe in their current neighborhood or having a close relationship with non-parental adults such as a grandmother had an important contribution to their capacity for resilience. Furthermore, these children neither agreed nor disagreed with the need to have been explained their parents' decision to divorce in order to be able to adjust and cope with their divorce, as shown by the score for statement 19.

Participants in the Factor 3 group highly ranked statements that signaled the importance of having a close relationship with peers and other non-parental adults in attempting to adjust and cope with a changing familial dynamic. Furthermore, they ranked statement 20 negatively, which indicated a disagreement with the statement that emphasizes the importance of having a transparent and supportive channel of communication between parents and the child in order to foster resilience.

For Factor 4, all six distinguishing statements proved to be significant at a *p*-value of <0.01 (Table 9). As expected of participants who loaded significantly on this factor, statements 4, 11, and 12 were ranked highest, signaling the importance of effective parental conflict styles within this group of children. Not only do the distinguishing statements of this factor emphasize effective conflict resolution strategies, but they also show a strong agreement that parents' ability to refrain from involving children in conflict situations has greatly aided children's capacity to adjust.

### 3.6. Consensus statements

Consensus statements refer to statements found among participants based on all four of the emerging factors. Hence, these statements do not distinguish between the different factors but show commonalities (Table 10).

**Table 10:** Consensus statements.

**Statement No**.	**Consensus statement**	**Factor 1**		**Factor 2**		**Factor 3**		**Factor 4**	
		***Q*** **-sort value**	***z*** **-score**	***Q*** **-sort value**	***z*** **-score**	***Q*** **-sort value**	***z*** **-score**	***Q*** **-sort value**	***z*** **-score**
3	My family loves me	3	1.6	2	0.6	1	0.56	2	1.04
7	I see my father regularly	2	1.41	1	0.59	1	0.8	1	0.58
21^*^	I believe I am able to achieve tasks at home and school	−1	−0.44	0	−0.29	0	0	0	0.07
34^*^	We stayed in the same neighborhood after the divorce	−3	−1.75	−3	−1.77	−3	−1.36	−3	1.77
35	I feel safe in my neighborhood	−2	−0.75	−3	−1.77	−1	−0.56	−2	−0.91
37^*^	My friends and I follow the rules	−2	−0.93	−2	−1.18	−2	−0.88	−2	−0.91
38^*^	I mostly have happy thoughts	−1	−0.69	−2	−0.89	−1	−0.8	−1	−0.23
40^*^	I enjoy laughing with friends and family	0	−0.03	−1	−0.3	−1	−0.56	−1	−0.53

For all listed statements, no significant differences were found between factors at a p-value of >0.01, and those flagged with a ^*^ are also non-significant at a p-value of >0.05.

Participants across the four factors agreed with statement 3 “My family loves me” and statement 7 “I see my father regularly,” which indicate a stable, loving familial environment, and frequent visitation with the non-custodial father as highly valued by all participants who participated in this study, irrespective of their ethnicity or age. On the other hand, statements that were most highly disagreed with across all four factors included statement 34 “We stayed in the same neighborhood after the divorce” and statement 35 “I feel safe in my neighborhood.”

## 4. Discussion

The current study examined factors affecting child resilience in Namibia, post-parental divorce from the perspective of the child. Using Q-Methodology, this research has identified several social-ecological systems within the child's environment that may protect children from harmful consequences associated with exposure to parental divorce. The current findings support the notion that, in the Namibian context, resilience cannot be limited to being defined as a trait but rather as an interactive process between different systems and how the child interacts with these systems. Children's resilience depends to a large extent on multiple systems and their capacity for nurturing resilience, especially within proximal systems such as the family unit and extended family members, the school, friends, and the wider community (Masten and Cicchetti, 2012).

A third of participants (33.3%) emphasized quality parent–child relationships as an important factor in their lives. These children's rankings indicated that they valued a close, secure relationship with either one or both parents before and after the divorce; they viewed their family environment as safe, stable, and loving and reported having regular visitation with their non-custodial parent. Masten (2014) states that children who live in environments rich in protective resources such as high-quality, supportive, and loving parent–child relationships tend to have an increased capacity for resilience. Rodgers and Rose (2002) found that the quality of parenting post-divorce plays the most crucial role in the adjustment and development of externalizing and internalizing behaviors among children. Fergus and Zimmerman (2005) and Lowenstein (2010) supported this and emphasized a secure attachment to either one or both parents as a mitigating influence and resilience-enhancing factor amidst exposure to a stressor, such as parental divorce. Given that systems are embedded and interconnected within each other, the capacity for resilience within a child might be reflective of the resilience capacity of the caregiving or family system (Masten and Palmer, 2019). This shows the importance of ensuring that both the parents' and the children's acute distress responses to the divorce do not merge and become chronic (Wallerstein, 1991) as it has the potential to become more complicated and challenging to recover from later on. Finally, while previous findings (Roehlkepartain and Syvertsen, 2014) recommend that parents maintain as much routine and familiarity post-divorce, participants loading on this factor did not emphasize this as an important feature. It may be that familial stability and quality parent–child relationships have been more influential in nurturing children belonging to this group and may have been a sufficient protective resource to combat the impact of a changed environment (whether it be a change in school, home, or neighborhood), which often accompanies a divorce.

The second most common factor grouping, with a third (33%) of participants loading, endorsed statements that emphasize effective conflict resolution between parents, including their ability to refrain from involving the child in conflict situations and maintaining a cooperative and civil relationship post-divorce. Although an increasing body of literature confirms that parental conflict after divorce increases the risk of poorer outcomes for the children involved (Sorek, 2020), including behavioral, emotional, and social difficulties (Johnston, 1994), there is conflicting evidence regarding whether potential maladjustment resulting from parental conflict should be attributed to conflict during the marriage or only after its dissolution (Elliott and Richards, 1991; Pryor and Rodgers, 2001). Nevertheless, ongoing conditions of conflict may cause children to resort to aligning with one parent against the other, feel compelled to sever the relationship with one parent, and experience subjective feelings of abandonment, heightened anxiety, and feeling “caught in the middle” (Kelly and Johnston, 2005). Therefore, it becomes imperative for parents to employ appropriate conflict resolution strategies in which children remain excluded from parental conflict as a means of increasing their chances for resilience post-parental divorce.

For the third grouping, participants loaded on statements that tapped into the wider community agreeing with statements emphasizing a healthy attachment to their school and its personnel. Numerous resilience-focused literature support this notion by identifying schools as a mesosystemic resource contributing to resilience for children exposed to adversity (Masten and Reed, 2002; Goldstein and Brooks, 2005; Harvey, 2007; Gentz et al., 2021). Findings from Hetherington and Elmore (2003) corroborate our findings showing that a secure attachment to their school tends to enable children affected by parental divorce to better cope with their new life circumstances. School environments defined by schedules and routines, along with the use of warm and consistent discipline, have been strongly associated with emotional and cognitive adjustment post-parental divorce (Hetherington and Elmore, 2003). Hence, children from unstable family circumstances greatly benefit from supportive school systems, teachers, and coaches (Hetherington and Elmore, 2003). According to a study conducted in Namibia, positive familial and school relationships have a higher influence on the wellbeing of children exposed to adversity such as violence, compared to factors such as individual child characteristics and poverty (Gentz et al., 2021).

Similarly, participants within the final factor (Factor 3) focused on their community, highly ranking statements related to healthy relationships with their grandparents, friends, and friends' parents. This is in unison with the collectivist values present in countries such as Namibia, which emphasize interconnectedness, interdependence, familial relationships, and social conformity (Santos et al., 2017). For example, research in Namibia has indicated the presence of informal systems of child care where extended family systems take over the care of children when they have lost one or both of their parents, either through death or through parental separation, as a deeply embedded practice particularly prevalent in rural areas (Brown et al., 2020). The role of the non-parental adult is also supported by Graber et al. (2016), who found that caring, non-parental adults and mentors play a significant role in promoting resilience among children exposed to parental divorce as they are able to provide children with needed support during this vulnerable period. Furthermore, findings from Akhtar et al. (2017) emphasize the quality of a close relationship between a child and their grandparents, and Sorek (2020) found that a close relationship with a grandparent may even buffer the child if there is parental conflict. In this case, it is a warm and close relationship that is important, and there is not necessarily a need to talk about the parental conflict. Grandparents have the potential to positively influence the overall wellbeing of children by providing them with an affectionate and supportive environment during a time characterized by emotional turmoil (Akhtar et al., 2017). Rankings by participants who loaded on this factor indicated a strong attachment to their peers as another important protective factor, which supports the increasing focus and importance placed on peer friendships and acceptance that starts to form during middle childhood. Informal social support networks such as relationships with peers provide the necessary communication and support imperative for healthy adjustment amidst parental divorce (Helgeson and Lopez, 2010). The children within this factor grouping did not agree that having a transparent and supportive channel of communication with their parents had been a significant protective factor in their attempts to cope with the divorce. In such instances, Graber et al. (2016) found that caring, non-parental adults and a secure social network equip children with the necessary protective resources to cope with their experiences.

Irrespective of the group/factor, all children across the four factors commonly emphasized a stable, loving familial environment, and frequent visitation with the non-custodial father. This highlights the potential role that regular contact with a non-custodial parent can contribute to children's post-divorce adjustment. This is supported by Lamb et al. (2005) whose research showed that children viewed the loss of regular contact with the non-custodial father as one of the saddest consequences of the divorce and expressed a desire for more time with their father. Indeed, researchers have suggested that the father–child relationship is just as important for emotional and behavioral adjustment as the mother–child relationship (Lowenstein, 2010).

It is imperative that children experience a sense of safety, stability, and peace within their family structure (Turner et al., 2012). Hence, if a divorced family is able to function well and provide and facilitate these core familial tasks, it holds the capacity to buffer against the impact of a reduced standard of living as well as facilitate the necessary emotional and social support in cases where parents need to take on more work and are therefore less physically and emotionally available to the needs of their children (Berger, 2002).

### 4.1. Practice recommendations

Our findings suggest that intervention strategies focusing on building resilience would be most beneficial if they focused on supporting or enhancing key protective factors. The parent microsystem remains pivotal in promoting resilience capacity in children. In light of this, it remains vital for parents to have access to support systems in the form of therapeutic support, education, family members, and the wider community, as well as access to alternative means of resolving conflict. Parents may receive valuable support from therapy in order to deal with their own sense of grief over the marriage as well as gain some valuable coping strategies and alternative, healthier communication patterns between them as parents. Another valuable avenue of support for parents is access to alternative means of resolving disputes, including the process of mediation and post-divorce counseling. By creating a healthy, productive post-divorce environment characterized by reduced parental conflict, parents can continue to meet the needs of their children more effectively and allow children to feel free to continue building a loving relationship with both parents (Carter, 2002). Furthermore, therapy aimed at challenging views and beliefs about divorce in both parents and their family members might be beneficial in situations where conservative views of divorce cause shame or guilt as well as reduced support from family and other community members. This is inclusive of a cooperative and supportive relationship between the parents with regard to visitations with the father (non-custodial parent), another commonly shared perspective among all participants.

The other two factors with significant loadings encompassed more community resources and reinforced the importance of contact with extended family support systems, schools, peers, and the wider community. These extra-familial individuals can potentially provide a source of stability and safety for children. These findings also point to the importance of ensuring children's time with friends and encouraging them to continue with extracurricular activities. Hence, as a recommendation for practitioners, therapeutic interventions that are directed toward the child, the parents, and the wider social support network will go a long way in promoting the necessary support required to promote healing and resilience (Masten, 2021).

School-based interventions also have the potential to nurture resilience in children exposed to adversity (Cefai et al., 2021). Such school-based resilience-enhancing initiatives are most successful when they encourage connectivity, learning, and are sensitive to diversity (Cefai et al., 2021). Furthermore, parents play a crucial role in promoting resilience within school children; hence, school-based interventions become more effective when supported by complementary home-based interventions (Weare and Nind, 2011). Participation by parents not only reinforces resilience competencies fostered at school but also helps to transfer these competencies into other contexts as well, including the home, peer groups, and the wider community.

Teachers play an integral part in promoting resilience among children coping with adversity such as parental divorce (Theron and Engelbrecht, 2012). Teachers play an active role in promoting positive outcomes among affected children through their daily presence within the child's microsystem, placing them in a favorable position to be able to impact child resilience (Theron and Engelbrecht, 2012). Supportive and caring teachers correlate with positive behavioral adjustment and academic success (Downey, 2008). Caring teachers who adopt an authoritative and consistent style of discipline and communicate and encourage attainable behavioral and scholastic expectations have been found to promote positive adjustment in children coping with parental divorce (Hetherington and Elmore, 2003). However, it is important for teachers to be acknowledged and receive the necessary training and support in order to be better equipped to engage with youth in an attempt to promote resilience amidst exposure to adversity (Theron and Engelbrecht, 2012). Furthermore, it is important for teachers to be sensitized to the contextual uniqueness of resilience in order for them to be able to understand and utilize the coping strategies of each child within their unique context (Theron and Engelbrecht, 2012), especially within a culturally diverse country such as Namibia. Finally, Cefai et al. (2021) emphasized the importance of ensuring that teachers' interpersonal needs and resilience are addressed in order to enable them to be emotionally, psychologically, and socially able to effectively attend to the social and emotional needs of their students.

### 4.2. Limitations and future research directions

Together with its contributions, this study has several limitations. The first limitation is the modest sample size and lack of diversity of participants. Only participants from within the Khomas Region in an urban area took part in the research, affecting the generalizability of our findings. Our reliance on a small, less representative sample was in part due to the complexity of obtaining post-divorce and resilient children. In addition, the outbreak of COVID-19 greatly influenced institutions' willingness to participate in the data collection process. A related limitation could be that the emotional impact and social restrictions that accompanied the pandemic may have influenced how the participants sorted the Q-statements, hence influencing the results of the study. Second, this study was conducted cross-sectionally, and that limits the researchers' capacity to adequately explore the complex interaction between the identified protective factors as well as the impact of time and pre-divorce conditions on both the identified protective factors and the children's capacity for resilience.

Future research may benefit from exploring the complex dynamics and resultant impact of siblings and stepparents on children's capacity for resilience. Furthermore, comparing the child's and both parents' perspectives on protective factors might provide significant insight into how these perspectives differ and influence how divorce and parenting are handled. The findings of this study should be corroborated by similar studies, in future, including longitudinal, pre–post-divorce studies in order to be able to assess the impact of circumstances before the divorce on children's post-divorce adjustment and their capacity for resilience.

## 5. Conclusion

Though divorce will continue to be a traumatic life transition for many Namibian children, it is evident that children have the potential to adjust and even thrive after such an experience provided that individual protective factors are present and they have access to valuable support, cohesion, and routine from both their family and the wider community. Furthermore, very little resilience research in general exists within the Namibian context. Such studies challenge the continued supremacy of Western research concerning resilience to decolonize knowledge surrounding this construct, and remove discourses that privilege a certain socioeconomic profile (Ungar, 2008), and recognize cultural practices and processes that may nurture resilience within unique contexts (Masten, 2021). Previous multi-country studies have found that resilience protective factors tend to be universal; their ranking in importance, their expression, and how they are used remain highly contextual and culture-specific (Grotberg, 1995; Gunnestad, 2006). For example, in African contexts, there seems to be a strong link between resilience, culture, and religion (Theron and Theron, 2010). Theron et al. (2013) went on to explain that the traditional African value of Ubuntu remains an imperative component of resilience from an Afrocentric perspective. Ubuntu refers to a collective way of living where an individual exists as part of the larger community. Values embedded in this concept include hospitality and mutual aid. Another typical value embedded within African families is interdependence among extended family members, which remains instrumental in nurturing resilience among African families (Dass-Brailsford, 2005). Evident in this study is the strong reliance on the community, including schools and peers as well as extended family members; however, open communication with parents is not as prominent as suggested by research from other contexts.

This study is also among the few that incorporated Q-Methodology in exploring the perspectives of children on positive factors that nurture their resilience amidst a parental divorce. It adds to the increasing literature recommending that children can be important actors in reflecting and reporting on their own lives (Ben-Arieh, 2005), including those from divorced families (Sorek, 2020).

Our findings emphasized the notion that resilience is not inherent to certain individuals and absent in others but rather involves thoughts, actions, and environmental resources that can be developed and utilized by anyone, even children (American Psychological Association, 2002). Furthermore, the findings highlighted the ecological nature of resilience by proving that the capacity for resilience depends on multiple systems and resources not only within the individual but also within their significant relationships with other systems within their unique environment (Masten, 2021). Family structure is indeed influential, but the most impactful characteristic of a family is how each member cares for the other (Turner et al., 2012). A well-functioning family structure exhibits the ability to meet specific needs presented during middle childhood. Among these are physical needs, such as food and shelter, and the need for positive peer and non-parental adult relationships; this is aided by parents choosing a good school and neighborhood and allowing frequent visitation with grandparents, friends, and the non-custodial parent.

## Data availability statement

The raw data supporting the conclusions of this article will be made available by the authors, without undue reservation.

## Ethics statement

The studies involving human participants were reviewed and approved by University of Namibia Research and Ethics Committee. Written informed consent to participate in this study was provided by the participants' legal guardian/next of kin.

## Author contributions

JV is the lead author, conceptualized the study, wrote the introduction, the method, and the results and discussion, and conducted the analysis with SG. SG conceptualized the study, wrote the introduction, the method, and the results and discussion with JV. All authors contributed to the manuscript and approved the submitted version.
